# Overlap Myositis in a Patient With Psoriasis Treated With Anti–TNF‐Alpha Therapy: A Case Report and Review of Literature

**DOI:** 10.1155/crrh/6133434

**Published:** 2026-08-28

**Authors:** Carlos H. Colegial, Paula V. Gaete

**Affiliations:** ^1^ Department of Pathology, Universidad Nacional De Colombia, Bogotá, Colombia, unal.edu.co; ^2^ Department of Neurology, Universidad Nacional De Colombia, Bogotá, Colombia, unal.edu.co

**Keywords:** anti-TNF, autoimmunity, case report, inflammatory myopathy, muscle, overlap myositis, tumor necrosis factor

## Abstract

Overlap myositis refers to the simultaneous occurrence of myositis and another systemic autoimmune disease. Association between psoriasis and inflammatory myopathy is uncommon. Tumor necrosis factor (TNF)‐alpha antagonists are used to treat patients with psoriasis and arthritis associated with psoriasis. Although the use of TNF‐alpha–blocking agents increases the risk of developing myopathy more than 4 times in people with psoriasis, this association is underrecognized. Here, we describe the case of a young male who developed inflammatory myopathy after the use of TNF‐alpha blocking therapy for the treatment of psoriasis, associated with cN‐1A and NXP2 autoantibodies. After cessation of the TNF‐alpha blocking agent and pulse corticosteroid therapy, the patient showed a good response, with increased muscle strength and decreased creatine kinase levels. This case highlights the importance of monitoring for muscle symptoms in patients treated with anti–TNF‐alpha agents and the heterogeneous clinical phenotypes that can occur with each autoantibody.

## 1. Introduction

Overlap myositis is part of the group of autoimmune inflammatory myopathies that also include dermatomyositis, immune‐mediated necrotizing myopathy, inclusion body myositis, and antisynthetase syndrome [1]. Overlap myositis refers to the simultaneous occurrence of myositis and another systemic autoimmune disease and accounts for more than 50% of autoimmune inflammatory myopathies. The most common associations in overlap myositis are systemic sclerosis, mixed connective tissue disease, systemic lupus erythematosus, and Sjogren syndrome [2].

Psoriasis is a chronic T‐cell–mediated skin disease that affects approximately 2% of the population [3]. Psoriasis is typically associated with seronegative arthritis and inflammatory bowel disease, while association between inflammatory myopathy is uncommon [4]. Tumor necrosis factor (TNF)‐alpha antagonists are used to treat patients with psoriasis and arthritis associated with psoriasis and have been associated with secondary autoimmune disorders. The use of TNF‐alpha–blocking agents increases the risk of developing myopathy in people with psoriasis by more than 4 times [4]. Here, we describe the case of a young male who developed inflammatory myopathy after the use of TNF‐alpha blocking therapy for the treatment of psoriasis.

## 2. Case Report

A 40‐year‐old male from Bogota, without a family history of autoimmune diseases and with a previous diagnosis of hypothyroidism, Type 2 diabetes mellitus, dyslipidemia, and psoriasis in treatment with high‐intensity statin therapy (atorvastatin 40 mg/day) and TNF‐alpha blocker (adalimumab), presented to the emergency department because of subacute proximal muscle weakness. Inflammatory myopathy was suspected because of a proximal decrease in muscle strength in all four extremities, not associated with cutaneous signs, muscle atrophy, or changes in reflexes. Statin therapy was suspended.

**FIGURE 1:**
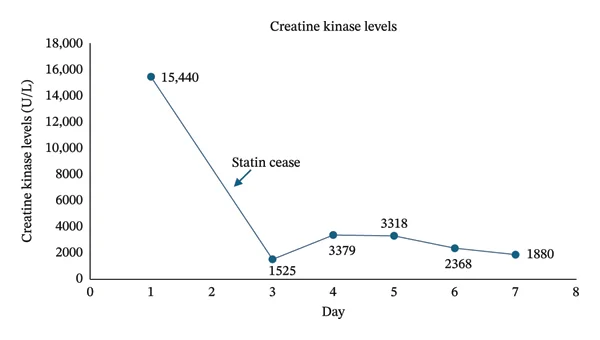
Change of creatine kinase (CK) values of the patient over the course of time.

Laboratory tests showed elevated transaminases (alanine transaminase 312 U/L, aspartate transaminase 191 U/L), lactic dehydrogenase (380 U/L), and creatine kinase (15440 U/L) as a sign of muscle turnover. Hemogram, serum electrolytes, and kidney function tests showed normal results (Table 1). Glycated hemoglobin was 6.37%. Initially, statin cessation decreased serum creatine kinase (Figure 1), but on the third day, an exacerbation of muscular weakness occurred, accompanied by signs of imminent respiratory failure. Thus, the patient was transferred to the intensive care unit.

**TABLE 1:** Initial laboratory tests.

Laboratory test	Result (reference range)
Leukocytes	10,010 (4500–11,000 cells/uL)
Neutrophils	6540 (2500–7000 cells/uL)
Lymphocytes	3250 (1000–4800 cells/uL)
Platelets	3,46,000 (1,50,000–4,50,000 cells/uL)
Hemoglobin	16.4 (13.2–16.6) g/dL
Ureic nitrogen	16 (8–24) mg/dL
Creatinine	0.54 (0.5–1.3) mg/dL
C‐reactive protein	0.13 (0–0.3) mg/dL
Glycated hemoglobin	6.37 (4–5.6)%
Calcium	9.5 (8.5–10.5) mg/dL
Potassium	3.7 (3.5–5.2) mEq/L
Sodium	143 (135–145) mEq/L
Magnesium	2 (1.3–2.1) mEq/L
Phosphorus	2.63 (2.5–4.5) mg/dL
Aspartate transaminase	191 (0–35) U/L
Alanine transaminase	312 (7–56) U/L
Lactic dehydrogenase	380 (105–233) U/L

**FIGURE 2:**
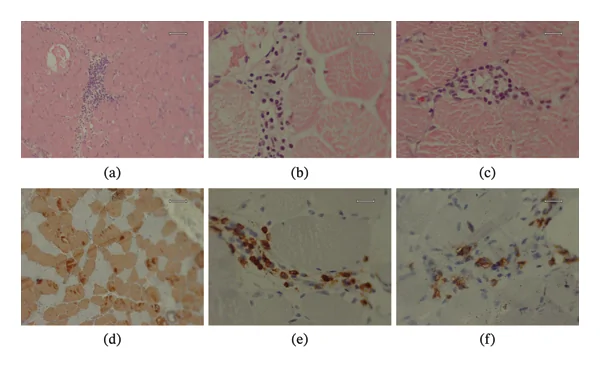
Muscle biopsy findings. Hematoxylin and eosin stain shows endomysial inflammatory infiltrate (a), perivascular inflammation (b), and necrotic myofiber phagocytosis (c). Fast myosin immunohistochemistry did not evidence a specific muscle fibers loss (d). CD‐8 (e) and CD‐20 (f) immunohistochemistry highlights the predominant *T* lymphocytic infiltrate.

**FIGURE 3:**
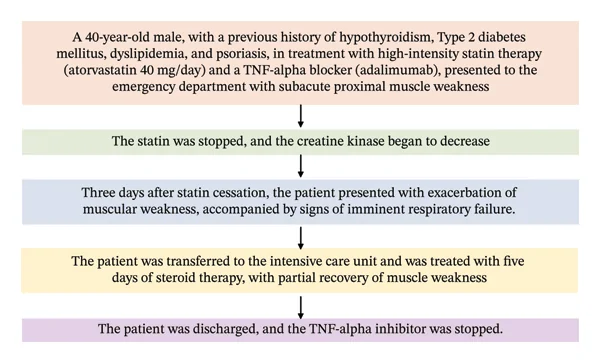
Clinical timeline.

A muscular contrast‐enhanced nuclear magnetic resonance imaging showed T2 hyperintensities in both thighs and arms, predominantly at the level of both adductor magnus, semitendinosus, semimembranosus, vastus medialis, biceps femoris, and triceps vastus lateralis, as a sign of muscle edema. A left triceps brachii muscle biopsy was taken, and pulse corticosteroid therapy was initiated. Neurophysiological studies showed normal sensory action potentials, polyphasic motor units, and decreased motor unit potential duration, consistent with myopathy. After five days of pulse corticosteroid therapy, the patient recovered some muscle strength; therefore, he was discharged with a rheumatology consultation with results of the histopathological study and the myositis‐associated antibodies. Myositis antibody panel showed positive NXP2 and cytosolic 5′‐nucleotidase 1A (cN‐1A) antibodies (Table 2), while histopathology studies showed perivascular inflammation with CD20‐ and CD8‐positive lymphocytes and phagocytosis of necrotic muscle fibers (Figure 2). Electron microscopy analysis did not evidence filamentous inclusions or rimmed vacuoles in muscle tissue. Overlap myositis was diagnosed, and anti–TNF‐alpha therapy was stopped. The patient continued his recovery with less muscle weakness in the 6‐month follow‐up. A clinical timeline is shown in Figure 3.

**TABLE 2:** Myositis auto‐antibodies panel.

Mi‐2a	Negative
Mi‐2b	Negative
TIF1‐gamma	Negative
MDA5	Negative
NXP2	Positive
SAE1	Negative
Ku	Negative
PM‐Scl100	Negative
PM‐Scl75	Negative
Jo‐1	Negative
SRP‐54	Negative
PL‐7	Negative
PL‐12	Negative
EJ	Negative
OJ	Negative
cN‐1A	Positive
Ro‐52	Negative

## 3. Discussion

Overlap myositis in the context of psoriasis is uncommon, although cases of dermatomyositis and antisynthetase syndrome in these patients had been previously reported [5–14]. A study made in Mayo Clinic from 1996 to 2011 found that the prevalence of myopathy in these patients was 1 in every 758 (0.13%). Median age at onset of psoriasis was 46.5 years, similar to the age of the patient described [4]. Inflammatory myopathy and psoriasis are conditions that share a common pathophysiology, including interferon‐alpha–induced responses and dysregulation of cytokines, such as TNF‐alpha. Some risk factors common to these entities are young age and a strong family history; 13% of patients with dermatomyositis also have a family history of psoriasis [8].

TNF‐alpha antagonists are an approved treatment for psoriasis [15] and are used in patients with dermatomyositis, reducing muscle and skin disease, including calcinosis, especially in refractory cases [16, 17]. Elevated levels of TNF‐alpha have been demonstrated in the serum of patients with dermatomyositis and within the calcium deposits [18]. Although some case series have suggested that TNF‐alpha blocking agents can also exacerbate muscle weakness [19] or trigger or exacerbate psoriasis [18, 20, 21].

Also, these medications have been associated with the induction of autoimmune phenomena [22]. Systemic lupus erythematosus and vasculitis are the most associated, together comprising 60% of cases of anti‐TNF‐alpha–induced autoimmune disease [19]. Myopathy induced by anti‐TNF accounts for less than 1% of reported cases of anti‐TNF–induced autoimmune phenomenon [23].

The increased risk of developing myopathy after treatment with TNF‐alpha antagonists has been outlined previously [13, 19, 23–26], with most patients improving their symptoms after cessation of TNF‐alpha blocking therapy [18]. Although clinical findings of myositis mostly regressed after the discontinuation of anti–TNF‐alpha therapy, cases of dermatomyositis with partial improvement in pulmonary findings have been described [24]; importantly, interstitial lung disease is also a complication of anti‐TNF therapy [22].

In a study of people with psoriasis and myopathy, patients exposed to TNF‐alpha antagonists were much more likely to develop myopathy than those who were not (OR = 4.45; 95% CI 1.41–14.0) [4]. The causality between inflammatory myopathy and anti–TNF‐alpha therapy can be described as not dose‐related and time‐delayed (range: 2 weeks to 34 months, mean: 12.7 months) [18].

The paradoxical onset of inflammatory myopathy in patients treated with TNF‐alpha blockers conflicts with the previously reported positive therapeutic effect of these agents. The mechanism underlying this paradoxical phenomenon remains elusive, but increased IFN‐gamma production after TNF‐alpha blockade might play a role, as IFN‐gamma is a key factor in the induction of dermatomyositis. Anti–TNF‐alpha therapy inhibits the cytotoxic T lymphocyte response that would normally suppress the autoreactive B‐cell response, thereby promoting humoral autoimmunity and increasing the Type I interferon system [18, 20, 21].

Anti–TNF‐alpha had also been associated with an increased risk of inflammatory myopathies, such as dermatomyositis and antisynthetase syndrome, in the context of other autoimmune diseases. Rheumatoid arthritis, more than other autoimmune diseases, increases the risk of dermatomyositis after anti‐TNF therapy [27]. Some risk factors described as increasing the risk of developing autoimmune phenomena during therapy with anti‐TNF antagonists include positivity for antinuclear antibody (ANA) or anti‐Jo1 antibodies [26]. These biomarkers should be measured before initiation of therapy, as the incidence of ANA positivity increases threefold with anti‐TNF therapy, even in the absence of a lupus‐like syndrome [28]. In patients with severe pulmonary, renal, or muscular involvement, cessation of anti–TNF‐alpha agents is recommended [22].

It is important to differentiate inflammatory myopathy in patients with psoriasis from other symptoms, such as myalgias. Musculoskeletal manifestations are present in approximately 30% of patients with psoriasis [29]. Inflammatory myopathies are characterized by proximal muscle weakness associated with elevated serum creatine kinase and myopathic changes on electromyography [1], as present in this case.

When inflammatory myopathy has been confirmed, biopsy and autoantibody testing can be useful for differentiating the exact type of overlap myositis. This is important because some inflammatory myopathies, like dermatomyositis, increase the risk of cancer [22], even in patients with previous autoimmune disease and anti–TNF‐alpha therapy; therefore, an extensive tumor screening must be performed. Cancer risk associated with cN‐1A and NXP2 autoantibodies is controversial. Most studies report a low probability of a paraneoplastic syndrome with cN‐1A autoantibodies [30]. However, NXP‐2 autoantibodies have been more associated with malignancy, including breast carcinoma, colorectal carcinoma, renal cell carcinoma, thyroid (papillary) carcinoma, lymphoma, myelodysplastic syndrome, chronic lymphocytic leukemia, endometrial carcinoma, lung carcinoma, nasopharyngeal carcinoma, neuroendocrine carcinoma, ovarian carcinoma, prostate carcinoma, thymic carcinoma, urothelial carcinoma, and Waldenström’s macroglobulinemia, with an OR of 4.2 ([95% CI 1.5–12.1], *p* = 0.008) [31]. Computed tomography of the thorax, abdomen, and pelvis, upper endoscopy, and colonoscopy were negative for malignancy in this patient.

Autoantibodies to cytosolic 5′‐nucleotidase 1A (cN‐1A) are classically associated with inclusion body myositis [32], with a specificity of 87%–100% [33]. However, recent studies have shown that these autoantibodies can also be present in other autoimmune diseases such as Sjogren syndrome, systemic sclerosis, systemic lupus erythematous, and psoriasis [34], and even in other inflammatory myopathies such as overlap myositis, antisynthetase syndrome, and dermatomyositis [35]. Considering the patient’s prior diagnosis of psoriasis and the absence of classic histopathologic findings of inclusion body myositis on muscle biopsy, the likelihood of this specific inflammatory myopathy is low.

In parallel, similar findings have been observed in patients with autoantibodies to NXP2, mainly being positive in patients with dermatomyositis, but recently also described in patients with Inclusion body myositis, overlap myositis, and antisynthetase syndrome [36]. The absence of cutaneous signs and calcinosis makes dermatomyositis less likely.

As previously described, overlap myositis is defined as the presence of myositis in the course of another connective tissue disorder. Muscle biopsy findings are heterogeneous, with necrosis and regeneration of myofibers and lymphocytic infiltration, mainly of CD4+ and CD8+ T cells, but also of CD19+ B cells, which are typical [37], as in this case.

In view of the patient’s clinical presentation, associated laboratory tests, and histopathologic findings, we consider this a case of overlap myositis with psoriasis and positive NXP‐2 and cN‐1A autoantibodies, associated with the use of TNF‐alpha–blocking agents. After cessation of the TNF‐blocking agent and pulse corticosteroid therapy, the patient showed a good response, with increased muscle strength and decreased creatine kinase levels. This case highlights the importance of monitoring muscle symptoms in patients treated with anti–TNF‐alpha agents and the heterogeneous clinical phenotypes that can occur with each autoantibody.

## Author Contributions

Both authors contributed to conceptualization, data curation, formal analysis, methodology, and writing.

## Funding

No funding was received for this manuscript.

## Ethics Statement

Ethical approval is not required, as this is a standard clinical case presentation and does not constitute formal clinical research.

## Consent

Written informed consent was obtained from the patient for publication of this case report and any accompanying images.

## Conflicts of Interest

The authors declare no conflicts of interest.

## Data Availability

The data that support the findings of this study are available from the corresponding author upon reasonable request.
